# Fusion of the dendritic cell-targeting chemokine MIP3α to melanoma antigen Gp100 in a therapeutic DNA vaccine significantly enhances immunogenicity and survival in a mouse melanoma model

**DOI:** 10.1186/s40425-016-0189-y

**Published:** 2016-12-20

**Authors:** James T. Gordy, Kun Luo, Hong Zhang, Arya Biragyn, Richard B. Markham

**Affiliations:** 1 The Department of Molecular Microbiology and Immunology, Johns Hopkins Bloomberg School of Public Health, 615 N. Wolfe Street, Baltimore, MD 21205 USA; 2 Immunoregulation Section, Laboratory of Molecular Biology and Immunology, National Institute on Aging, National Institutes of Health, 251 Bayview, Blvd, Suite 100, Baltimore, MD 21224 USA

**Keywords:** DNA Vaccine, MIP3α, MIP3alpha, or CCL20, B16 Melanoma, Gp100, Therapeutic cancer vaccine, Chemokine-antigen fusion, In vivo electroporation

## Abstract

**Background:**

Although therapeutic cancer vaccines have been mostly disappointing in the clinic, the advent of novel immunotherapies and the future promise of neoantigen-based therapies have created the need for new vaccine modalities that can easily adapt to current and future developments in cancer immunotherapy. One such novel platform is a DNA vaccine fusing the chemokine Macrophage Inflammatory Protein-3α (MIP-3α) to an antigen, here melanoma antigen gp100. Previous published work has indicated that MIP-3α targets nascent peptides to immature dendritic cells, leading to processing by class I and II MHC pathways. This platform has shown enhanced efficacy in prophylactic melanoma and therapeutic lymphoma model systems.

**Methods:**

The B16F10 melanoma syngeneic mouse model system was utilized, with a standard therapeutic protocol: challenge with lethal dose of B16F10 cells (5 × 10^4^) on day 0 and then vaccinate by intramuscular electroporation with 50 μg plasmid on days three, 10, and 17. Efficacy was assessed by analysis of tumor burden, tumor growth, and mouse survival, using the statistical tests ANOVA, mixed effects regression, and log-rank, respectively. Immunogenicity was assessed by ELISA and flow cytometric methods, including intracellular cytokine staining to assess vaccine-specific T-cell responses, all tested by ANOVA.

**Results:**

We demonstrate that the addition of MIP3α to gp100 significantly enhances systemic anti-gp100 immunological parameters. Further, chemokine-fusion vaccine therapy significantly reduces tumor burden, slows tumor growth, and enhances mouse overall survival compared to antigen-only, irrelevant-antigen, and mock vaccines, with efficacy mediated by both CD4+ and CD8+ effector T cells. Antigen-only, irrelevant-antigen, and chemokine-fusion vaccines elicit significantly higher and similar CD4+ and CD8+ tumor-infiltrating lymphocyte (TIL) levels compared to mock vaccine. However, vaccine-specific CD8+ TILs are significantly higher in the chemokine-fusion vaccine group, indicating that the critical step induced by the fusion vaccine construct is the enhancement of vaccine-specific T-cell effectors.

**Conclusions:**

The current study shows that fusion of MIP3α to melanoma antigen gp100 enhances the immunogenicity and efficacy of a DNA vaccine in a therapeutic B16F10 mouse melanoma model. This study analyzes an adaptable and easily produced MIP3α-antigen modular vaccine platform that could lend itself to a variety of functionalities, including combination treatments and neoantigen vaccination in the pursuit of personalized cancer therapy.

**Electronic supplementary material:**

The online version of this article (doi:10.1186/s40425-016-0189-y) contains supplementary material, which is available to authorized users.

## Background

The recent therapeutic success of immunotherapies [1] and the identification of cancer neoantigens as potential therapeutic targets [2, 3] have generated renewed interest in the field of cancer vaccines. Although only one therapeutic cancer vaccine is currently FDA-approved (Sipuleucel-T [4]), hypothesized synergies between current and future immunotherapies [5] have increased the need for new vaccine platforms that can best address the new immunotherapeutic opportunities.

DNA vaccines offer many advantages as cancer therapies. They generate effector immunity from all three arms of the adaptive immune response, particularly including CD8+ T-cells [6]. They avoid the inclusion of extraneous and possible deleterious antigens that may be components of bacterial or viral-based vaccines [6]. They stimulate innate immunity and avoid issues of safety and practicality associated with various vectors [6]. They can also be readily adapted to novel or mutating antigenic targets, are stable at room temperature, and can be constructed quickly [6]. Clinical trials with a variety of antigens have demonstrated safety and immunogenicity of clinical DNA vaccines [7, 8]. However, initial trials for therapeutic DNA cancer vaccines have all shown limited effectiveness [9]. More recent advances in DNA vaccination modalities have rekindled interest in their potential efficacy for cancer therapy [10, 11]. Of note, DNA vaccines have shown efficacy in animals, with three licensed for veterinary use [12–14].

One of the primary hurdles for DNA vaccines has been their limited potency in the clinical setting [6]. Novel approaches to in vivo DNA delivery are being developed to address this issue. In vivo electroporation has been shown in animal models to enhance the breadth and potency of elicited immune responses [15–18]. Mechanistic studies have shown electroporation increases DNA uptake, stimulates local inflammation at the vaccination site, and enhances amount of vaccine antigen produced in situ [19–21]. In vivo electroporation is currently being utilized in the veterinary clinic as a mode of introducing a hormone into pregnant sows [22] and is currently undergoing clinical trials [23, 24].

Additionally, investigators have been taking advantage of the inherent flexibility of DNA to add immunomodulators to the vaccine construct in order to enhance the efficiency of initiating a specific immune response. Many studies have focused on increasing productive contact of nascent vaccine antigens to antigen presenting cells (APCs), especially dendritic cells (DCs). One approach is to fuse antigens to cytokines such as GM-CSF that can stimulate the development, proliferation, and maturation of DCs and monocytes [25–27] or to chemokines like CCL5 [28], CCL19 [29], MIP3α (also known as CCL20) [30–35], or other molecules [36–40] that can recruit and/or target nascent peptides to APCs. MIP3α fusion vaccines have been shown to direct antigen to immature DCs via CCR6 and mediate antigen uptake in a fusion dependent manner [39], after which, antigens are cross presented by both MHC class I and II, activating significant responses from both CD4+ and CD8+ T cells [31–33].

In the current studies, a DNA vaccine administered by intramuscular electroporation with a construct fusing MIP3α to the melanoma tumor-associated antigen gp100 has been analyzed in a therapeutic vaccination protocol utilizing the B16F10 melanoma mouse model system. MIP3α-antigen fusion DNA vaccine constructs have shown efficacy in a prophylactic melanoma model against gp100 [33], a therapeutic lymphoma model against oncofetal antigen (OFA) [31], and a prophylactic malaria model against circumsporozoite protein (CSP) [30]. Here we compare therapeutic MIP3α-gp100 vaccination to a construct with a mutated MIP3α sequence that abrogates its function, effectively providing a gp100 antigen-only vaccine, and to a construct fusing the chemokine to an antigen irrelevant to this system, CSP. These experiments show that inclusion of functional MIP3α in the vaccine construct used in the therapeutic protocol enhances immunogenicity, slows tumor growth, and significantly extends survival compared to antigen-only and irrelevant-antigen vaccinations.

## Methods

### Animals and tumor model

Five to six week old female C57BL/6 (H-2b) mice were purchased from Charles River Laboratories (Wilmington, MA) and maintained in a pathogen-free micro-isolation facility in accordance with the National Institutes of Health guidelines for the humane use of laboratory animals. All experimental procedures involving mice were approved by the IACUC of the Johns Hopkins University (Protocol number MO13H219 and MO16H85). B16F10 mouse melanoma cells were a generous gift from Dr. Arya Biragyn (NIH, Baltimore, MD). Six to eight week old mice were challenged in the left flank subcutaneously with a lethal dose (5 × 10^4^ cells) of B16F10 melanoma. Tumor size was recorded as square mm, representing tumor length × width (opposing axes) measured by calipers every 1–3 days. Mice were kept in the study until one of the following occurred: mouse death, tumor size eclipsing 20 mm in any direction, or extensive tumor necrosis resulting in excessive bleeding.

### Plasmids and vaccination

Vaccine consisted of purified plasmid DNA in endotoxin-free PBS. The plasmid encoded either MIP3α-gp100, MIP3α-CSP as described [30], or dMIP3α-gp100 fusion sequence as described [33]. dMIP3α-gp100 vaccine DNA is identical except for a point mutation in the chemokine changing a structurally necessary cysteine to serine (C6S), which abrogates chemokine functionality [33]. Vaccination plasmid was extracted from *E. coli* using Qiagen® (Germantown, Md) EndoFree® Plasmid Maxi and Giga Kits. Vaccine DNA purity, quality, and quantity were verified by gel electrophoresis, restriction enzyme analysis, Nanodrop® spectrophotometry, and full insert sequencing. Mock vaccinations comprised of endotoxin-free PBS only. DNA injections were administered into the hind leg tibialis muscle. Immediately following injection, the muscle was pulsed using an ECM 830 Electro Square Porator™ with 2-Needle Array™ Electrode (BTX Harvard Apparatus®; Holliston, MA) under the following parameters: 106 V; 20 ms pulse length; 200 ms pulse interval; 8 total pulses. Vaccinations of 50ug/dose were delivered at days noted in figure legends. Prophylactic efficacy of the vaccine was confirmed, replicating previously reported results in which DNA was delivered by gene gun [33] [Additional file 1]. Vaccine DNA was also confirmed to express specific protein after transfection into Hek-293 T cells [Additional file 2], as detected by Western blot analysis using antibodies targeting the myc tag present at the 3′ end of the construct. As described by others, vaccination for the therapeutic model began on day three [41, 42].

### In cell ELISA

Humoral immune responses to the vaccine were tested by an In-Cell ELISA assay to detect overall response to native B16F10 proteins, including gp100. The studies utilized the standard protocol for In-Cell ELISA from Abcam® (Cambridge, UK). In brief, wells of tissue-culture treated 96-well plates were seeded with 5 × 10^4^ B16F10 cells and were allowed to adhere for 3–4 h at 37 °C. Adherence was verified by microscopy before proceeding. Cells were fixed, incubated with serum or primary control antibody (rabbit anti-gp100 ab137078 [Abcam, Inc.; Cambridge, UK]) at varying dilutions overnight at 4 °C, blocked with 2% BSA, and then incubated with peroxidase-conjugated goat anti-mouse IgG (serum) or goat anti-rabbit IgG(control) (Jackson ImmunoResearch Laboratories, West Grove, PA) at a dilution of 1:5000. Wells were developed for 1 h using ABTS® ELISA HRP Substrate (KPL, Gaithersburg, MD). The data were collected using the Synergy™ HT (BioTek Instruments, Inc. Winooski, VT).

### Extraction of splenocytes and TILs

Spleen and tumor cell suspensions were prepared by grinding sterile excised tissue between the frosted ends of microscope slides and then passing the tissue through a sterile 60 μM mesh. Splenocytes were processed by lysing red blood cells and washing with sterile PBS. Tumor lysate was washed with sterile PBS, and tumor-infiltrating lymphocyte (TIL) fraction was enriched by Lympholyte®-M Cell Separation Media (Cedarlane®, Burlington, NC) according to the manufacturer’s protocol. Prior to use all cells were counted by a Z1™ Coulter Counter® (Beckman Coulter, Inc.; Brea, CA) and/or a hemocytometer with Gibco™ Trypan Blue solution 0.4% (Life Technologies, Carlsbad, CA).

### Intracellular cytokine staining and flow cytometry

Enriched splenocytes or TILs were seeded onto Falcon® Multiwell 24-well tissue culture treated plates (Corning, Inc.; Corning, NY) at 1 × 10^6^ cells per well (or all cells if total is less) and stimulated for 3–4 h at 37 °C with known immunodominant gp100_25-33_ (KVPRNQDWL) peptide or control HA (YPYDVPDYA) peptide (JHU School of Medicine Synthesis & Sequencing Facility; Baltimore, MD) combined with Protein Transport Inhibitor Cocktail and costimulatory anti-CD28 and anti-CD49d agonizing antibodies (eBioscience, Inc. San Diego, Ca). Cells were collected, washed, fixed, permeabilized, and stained using standard laboratory protocols for intracellular staining. Fixation and permeabilization buffers from Mouse Regulatory T Cell Staining Kit #2 (eBioscience, Inc. San Diego, Ca) were used. Stains utilized were the following anti-mouse mAbs: PercPCy5.5 conjugated anti-CD3, APC-conjugated anti-IFNγ, FITC-conjugated anti-CD8, and PE-conjugated anti-CD4 (eBioscience, Inc. San Diego, CA). Utilized FACSCalibur™ and LSRII™ Flow Cytometers (BD Biosciences, San Jose, CA). Flow Data analyzed by FlowJo Software (FlowJo, LLC Ashland, OR).

### Lymphocyte depletion

To deplete the CD4+, CD8+, or both T cell subsets, immunized mice were injected i.p. with anti-CD4 (GK1.5), anti-CD8 (2.43), or both mAbs, which were generous gifts from Dr. Fidel Zavala (JHSPH, Baltimore, MD). Negative control vaccinated mice received isotype Rat IgG2b antibody against KLH (LTF-2) purchased from BioXCell (West Lebanon, NH). 100 μg of antibody was given to each mouse i.p. on days -1, 0, and 7 from tumor challenge. Depletion efficacy was tested on days 0 and 8 or 10 by two-color flow cytometry analysis of peripheral blood lymphocytes using a FACSCalibur™ cytometer (BD Biosciences, San Jose, Ca) with FITC conjugated anti-mouse CD4 and APC-conjugated anti-mouse CD8 mAbs (eBioscience, Inc. San Diego, Ca).

### Statistics and availability of data

Tumor size, immunologic, and flow cytometric analyses were statistically tested by one-way ANOVA with Bonferonni correction and/or Tukey’s multiple comparisons test. Mouse survival studies were statistically tested by the log-rank test. Tumor time course regressions were analyzed by mixed effect regression models. STATA v11.2 (StataCorp, College Station, TX) and Prism 6 (GraphPad Software, Inc. San Diego, CA) were utilized for statistical analyses and figure creation. Significance level of α ≤ 0.05 was set for all experiments. The dataset supporting the conclusions of this article is included within the article’s additional files [Additional file 3].

## Results

### Systemic immune response

**Fig. 1:**
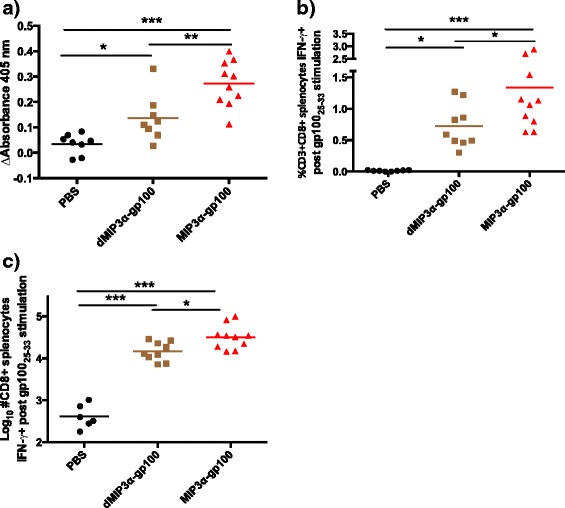
Systemic immune parameters of vaccine groups in prophylactic vaccination setting. Mice were vaccinated three times at 1 week intervals with endotoxin-free PBS, dMIP3α-gp100, and MIP3α-gp100 fusion vaccine. Analysis occurred 2 weeks post third vaccination. Data represent two independent experiments with 3–5 mice per group per experiment. **a** Analysis of relative antibody production against B16F10 cells. In-Cell ELISA performed utilizing fixed B16F10 cells as antigens. Experimental data are shown at a 1:2000 serum dilution after 30-min colorimetric development. Absorbance values from pre-immune mice were subtracted from post immune mice to obtain the delta absorbance. All groups were significantly different from each other by ANOVA. **b-c** Analysis of splenic CD8+ T cells reactive to ex vivo stimulation by gp100_25-33_ peptide. Activation was signaled by cytoplasmic IFN-γ accumulation as measured by Intracellular Cytokine Staining Flow Cytometry. Panel b shows the data as percentage of CD3+ splenocytes. Panel c estimates the total number of reactive CD3 + CD8+ splenocytes by extrapolating flow cytometric data to measured splenic total cell counts. For both panels, all groups differ significantly from each other, as determined by by ANOVA, *p < 0.05, **p < 0.01, ***p < 0.001

To initially evaluate the immunogenicity of the DNA construct, systemic immune parameters were examined. Mice were vaccinated three times at 1 week intervals and then analyzed 2 weeks after the third immunization. The MIP3α-gp100 vaccine elicited significantly higher levels of B16F10-specific antibodies than antigen-only vaccine, denoted as dMIP3α-gp100 (*p* = 0.004), and mock vaccine (*p* < 0.001; Fig. 1). Interestingly, antigen-only vaccine had significantly higher B16F10-specific antibody levels than mock vaccine (*p* = 0.044).

As was the case for the antibody concentration, the antigen-only vaccine elicited a moderate vaccine-specific CD8+ T-cell response that significantly differed from the mock vaccination by both percentage (*p* = 0.030; Fig. 1b) and total number (*p* < 0.001; Fig. 1c) of CD8+ T cells reactive to the immunogenic gp100_25-33_ peptide. The addition of MIP3α to the vaccine significantly increased the percentage of (*p* = 0.049) and total number of (*p* = 0.026) vaccine induced CD8+ T cells compared to the antigen only vaccine, increasing the CD8+ T cell numbers by 46% (Fig. 1b-c). The MIP3α-gp100 vaccine elicited significantly higher percentages and numbers of vaccine-specific CD8+ T cells compared to mock vaccination (*p* < 0.001 for both; Fig. 1b-c).

### Therapeutic vaccination model

**Fig. 2:**
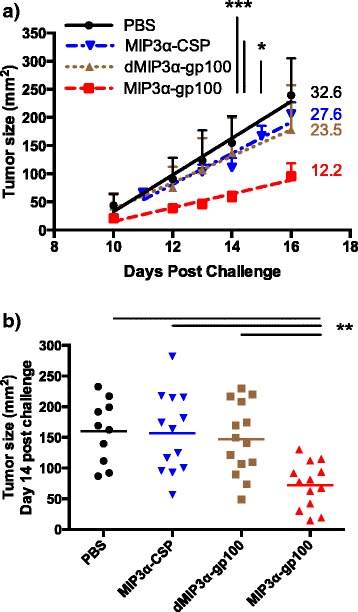
Vaccine effects on tumor growth in therapeutic model. Vaccinations occurred on days 3, 10, and 17 post challenge. **a** Tumor growth rate was assessed between days 10 and 16, day 10 being the point at which tumor growth of the negative control group began accelerating and day 16 being the point at which mice began to be censored due to endpoints being reached. The graph shows one representative experiment of two, five to seven mice per group and includes linear regression lines and slopes. The slope of tumor growth among recipients of MIP3α-gp100 vaccine differed significantly from dMIP3α-gp100, MIP3α-CSP, and mock PBS vaccination, as evaluated using a statistical mixed effects regression model. The groups receiving dMIP3α-gp100 and MIP3α-CSP did not differ significantly compared to each other or to the group receiving mock vaccination. Error bars represent standard error. **b** Tumor size at day 14 post challenge, the last point before any mice were removed from experiments. The data are representative of two experiments, with 5–8 mice per group per experiment. MIP3α-gp100 vaccine recipients had significantly smaller tumors compared to dMIP3α-gp100, MIP3α-CSP, and mock PBS vaccinated mice, as determined by ANOVA. dMIP3α-gp100 and MIP3α-CSP were not significantly different from each other or from mock. *p < 0.05, **p < 0.01, ***p < 0.001

The potential of this vaccine construct to be utilized in a therapeutic setting against a solid tumor was assessed, comparing MIP3α-gp100 vaccination to dMIP3α-gp100, MIP3α-CSP, and PBS vaccines. A therapeutic regimen was developed with mice vaccinated on days three, 10, and 17 post challenge with a lethal dose of B16F10 cells. Utilizing statistical mixed effects regression models, it was determined that the overall slope of the tumor growth regression line was reduced in the MIP3α-gp100 vaccinated group compared to the antigen-only vaccinated group by 48% (*p* = 0.029), to the irrelevant-antigen group by 56% (*p* < 0.001), and to the mock vaccine group by 63% (*p* < 0.001), whereas mock, antigen-only, and irrelevant-antigen vaccines showed no significant differences to each other in slope (Fig. 2a). Slower overall growth also provides evidence that the differences seen in these experiments are not due to blocks to tumor transplantation.

**Fig. 3:**
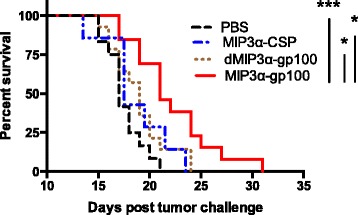
Vaccine effects on mouse survival in a therapeutic model. Vaccinations occurred on days 3, 10, and 17 post challenge. Mice were removed from the study at the following endpoints: death, tumor size surpassing 2 cm in any dimension, or excessive tumor bleeding and ulceration. Data representative of two experiments, 5–8 mice per group per experiment. Mice in the MIP3α-gp100 vaccine group exhibit significantly enhanced survival compared to the dMIP3α-gp100, MIP3α-CSP, and mock PBS vaccination groups by the log-rank test. dMIP3α-gp100 and MIP3α-CSP did not differ significantly from each other or from mock. *p < 0.05, **p < 0.01, ***p < 0.001

In addition to tumor growth, the tumor burden of MIP3α-gp100 vaccine recipients proved to be significantly lower than mock vaccination at most time-points tested and significantly lower than antigen only vaccine on the critical day 14 time-point – the last time point before any mice were removed from the study. On day 14 post challenge, the average tumor size was reduced in the MIP3α-gp100 group by 51% compared to the antigen-only group (*p* = 0.004), by 54% compared to irrelevant-antigen group (*p* = 0.001), and by 55% compared to the mock group (*p* = 0.001; Fig. 2b). Survival analysis mirrored tumor growth and burden analyses. MIP3α-gp100 vaccination significantly enhanced survival as compared to antigen-only (*p* = 0.017), irrelevant-antigen (*p* = 0.021) and mock (*p* < 0.001) vaccines. MIP3α-gp100 vaccination enhanced median survival by 10%, 24% and 24% compared to antigen-only, irrelevant-antigen, and mock vaccinations, respectively (Fig. 3). Antigen-only, irrelevant-antigen, and mock vaccinations did not have significantly different survival curves compared to each other (Fig. 3).

### T-cell subset depletion

**Fig. 4:**
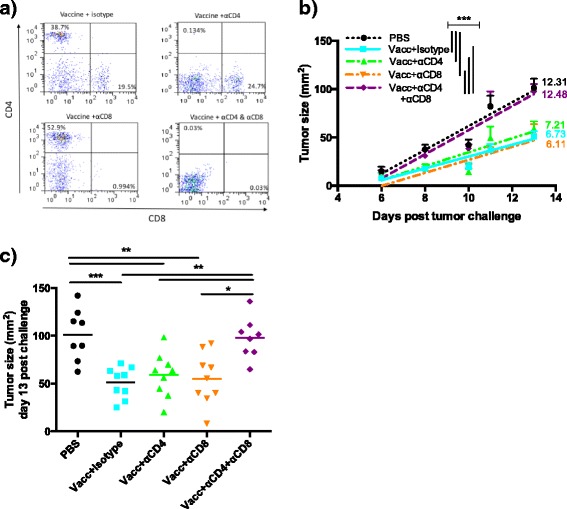
Vaccine effector T-cell responses analyzed by subset depletions. Mice were vaccinated three times at 1 week intervals and then challenged with a lethal dose (5 × 10^4^) of B16F10 cells. T-cell subsets were depleted one day prior to challenge, on day of challenge, and 7 days post challenge. Quality of the depletions was assessed by flow cytometric analysis of peripheral blood lymphocytes on days 0 and 8 or 10. **a** Representative flow cytometry plots shown depicting CD4 and CD8 expression gated on overall lymphocytes from blood collected at day 10 post challenge. **b** Tumor growth regression plot from day 6 to 13 post challenge, assessed by mixed effects regression. **c** Tumor size at day 13 post challenge, with significance assessed by ANOVA. All data are from two independent experiments of 4–5 mice per group. *p < 0.05, **p < 0.01, ***p < 0.001

To determine if effector T-cells played a role in mediating this enhanced protection, and, if so, which subsets might be involved, groups of mice were vaccinated three times over 3 weeks to develop vaccine-specific effector responses and then were challenged with tumor under differing depletion conditions: depleting CD4+, CD8+, both CD4+ and CD8+, and no depletion of T cells. Figure 4a shows representative flow cytometric analysis of depletion efficacy. In a mouse lymphoma model, a similar MIP3α-OFA vaccine showed the CD8+ T-cell effector response to be essential for protection with the CD4+ T-cell effector response being expendable [31]. However, in this melanoma solid tumor model, depleting CD4+ or CD8+ T-cells individually show a similar phenotype as the isotype depletion control. Single depletions have similar tumor growth rates and tumor sizes compared to isotype depletion (Fig. 4b-c). Importantly, depleting both subsets of T-cells simultaneously provided a phenotype similar to the unvaccinated control in those same analyses (Fig. 4b-c).

### Tumor infiltrating lymphocytes

**Fig. 5:**
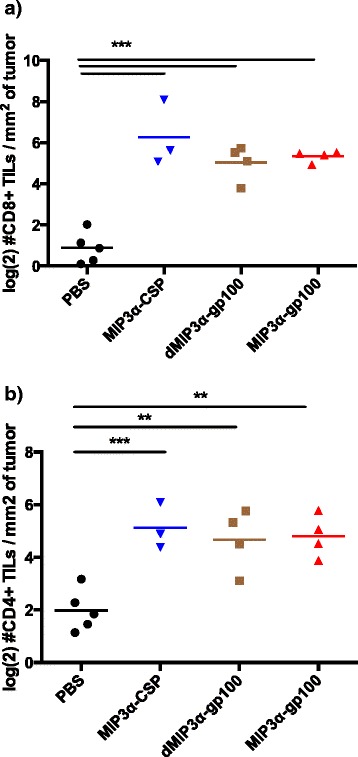
Vaccine effects on tumor infiltrating lymphocytes (TILs). Vaccinations occurred on days 3 and 10 post challenge. Mice were sacrificed on day 17 and lymphocyte-enriched tumor suspensions were analyzed by flow cytometry. **a** shows CD8+ TILs and **b** CD4+ TILs. Data show one representative experiment with 3–5 mice per group. Two independent experiments were performed. All three vaccine formulations have significantly higher CD4 and CD8 TILs compared to mock vaccination but not to each other, as assessed by ANOVA. *p < 0.05, **p < 0.01, ***p < 0.001

It has been documented that presence and activity of tumor infiltrating lymphocytes (TILs) can correlate with anti-tumor responses in melanoma patients [43]. The intratumoral characteristics of MIP3α-antigen vaccine responses have not previously been documented. Utilizing a therapeutic vaccination protocol as outlined, total CD4+ and CD8+ TILs were harvested 1 week after the second vaccination, counted by flow cytometry, and normalized by tumor size. Surprisingly, MIP3α-gp100, irrelevant-antigen, and antigen-only vaccines all induced significantly higher CD8+ TIL (all *p* < 0.001) and CD4+ TIL (all *p* < 0.01) responses compared to mock vaccine and were at similar levels to each other (Fig. 5a-b). However, antigen-only and irrelevant-antigen vaccines did not provide clinically relevant responses, not differing significantly from the negative control group in tumor growth, size, and survival (Figs. 2 and 3). In this system, TIL levels themselves appear not to correlate with protection. In addition, vaccine did not significantly alter levels of tumor-infiltrating CD25 + Foxp3+ regulatory CD4+ T-cells (Data not shown).

**Fig. 6:**
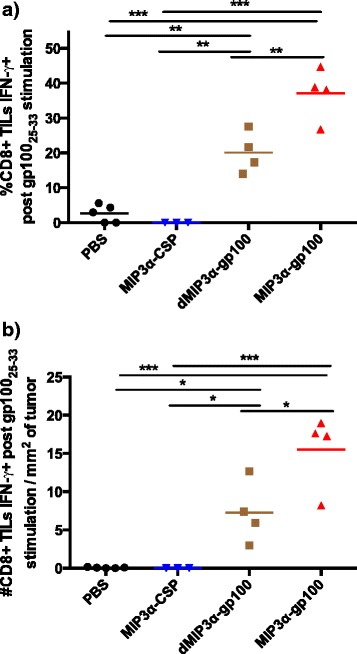
Vaccine-specific CD8+ T-cell tumor infiltrate analysis. Vaccinations occurred on days 3 and 10 post challenge. Mice were sacrificed on day 17 and lymphocyte-enriched tumor suspensions were collected. CD8+ TILs reactive to ex vivo stimulation by gp100_25-33_ peptide were delineated by Intracellular Cytokine Staining Flow Cytometry measuring cytoplasmic IFN-γ accumulation post stimulation. **a** Percentage of CD8+ TILs reactive to antigen. **b** Estimated total number of reactive CD8+ TILs normalized to tumor size. All groups were significantly different from each other by ANOVA except for the comparison of PBS to MIP3α-CSP. HA irrelevant negative peptide and PMA/ionomycin positive controls confirmed the protocol validity (data not shown). Data are from one of two representative experiments with 3–5 mice per group. *p < 0.05, **p < 0.01, ***p < 0.001

Finally, the levels of CD8+ TILs that secrete IFN-γ upon stimulation with immunodominant gp100_25-33_ vaccine antigen were assessed. Antigen-only vaccine induced a moderate response, with significantly higher gp100_25-33_-reactive CD8+ TILs by percentage (*p* < 0.01) and normalized total numbers (*p* < 0.05) compared to the PBS and irrelevant-antigen vaccinated negative control groups (Fig. 6a-b). MIP3α-gp100 vaccination significantly enhanced the percentage (*p* < 0.01) and normalized numbers (*p* < 0.05) of gp100_25-33_-reactive CD8+ TILs compared to antigen-only vaccine and compared to irrelevant-antigen (*p* < 0.001) and mock (*p* < 0.001). Although the two vaccines elicit a similar number of total TILs, the MIP3α-gp100 vaccine elicits a more robust vaccine-specific effector TIL response that correlates with the enhancement of tumor suppression and mouse survival seen.

## Discussion

Our data demonstrate that the addition of the chemokine MIP3α to the gp100 DNA vaccine construct enhanced vaccine immunogenicity and therapeutic potential. Although the antigen-only vaccine elicited a significant anti-gp100 immune response compared to the mock vaccine, when utilized as a therapy, only the MIP3α-gp100 vaccine slowed tumor growth and enhanced mouse survival. Further, MIP3α fused to irrelevant antigen CSP showed no anti-tumor activity, despite the previously demonstrated ability of the CSP construct to function as a highly efficacious vaccine for preventing malaria in a mouse model system [30]. Previous studies have conclusively shown that MIP3α must be fused to its antigen in order to enhance immunogenicity [39].

As has been shown in vitro [32, 33], MIP3α-gp100 vaccine directs the antigen in such a way that both CD4+ and CD8+ effector T-cells can be activated. In this study, T-cells were depleted after a prophylactic vaccination regimen in order to selectively deplete vaccine-specific effector cells and not disturb the immune activation phase of the vaccine response. If CD4+ T-cells were depleted in a therapeutic study, one would not know if the effect was due to lack of CD4+ anti-tumor effector response or due to lack of CD4+ T-cell help in the activation of a vaccine-specific CD8+ T-cell response.

Depletion of either the CD4+ or CD8+ effector T-cell population showed a protection phenotype similar to the non-depleted vaccine group, while depletion of both led to no protection, similar to that observed with mock vaccination. The lack of protection seen in the double depletion group provides evidence that antibodies elicited by the vaccine do not provide significant anti-tumor immunity on their own. Large tumor size outliers in both single depletion groups suggest that some proportion of the mice are reliant on the depleted subset for protection, but the overall groups either utilize both effector subsets relatively equally or one is able to compensate for lack of the other when necessary. The roles and mechanisms of tumor infiltrating effector CD4+ TILs are complex and still being defined [44], and therefore the intriguing finding of effector CD4+ T cells providing therapeutic efficacy in the absence of CD8+ T cells will be the subject of future work.

Finally, the data show that the therapeutic protection phenotype provided by MIP3α did not correlate with overall TILs, but did correlate with gp100_25-33_ vaccine peptide-reactive CD8+ TILs, elucidating that the immune activity of and not the quantity of the TILs correlates with therapeutic efficacy.

Vaccine efficacy depends on identification of appropriate target antigens, deliverance of those antigens in a form that elicits a relevant immune response, administration of vaccine by a route that brings it into contact with the critical immune cells, and selection of effective adjuvants/immunomodulators. For DNA vaccines, addition of MIP3α to circumsporozoite protein (CSP) with vaxfectin adjuvant [30] creates a robust, protective antibody response against malaria, addition of MIP3α to oncofetal antigen (OFA) given by gene gun creates a therapeutic response against lymphoma mediated by CD8+ T-cells [31], and as reported here, addition of MIP3α to gp100 given by intramuscular electroporation creates a therapeutic response against melanoma mediated by both CD4+ and CD8+ effector T-cells. All of these experiments have shown responses to be significantly enhanced by the chemokine in different contexts. Co-administration of MIP3α can enhance vaccine responses by enhanced DC recruitment [45]. However, our previous studies have indicated that in the context of a DNA fusion vaccine, MIP3α is acting by directing nascent expressed protein antigens to DCs, not by recruiting DCs in vivo [30]. Therefore, we hypothesize that in this context, the pro-inflammatory response elicited by electroporation serves as the adjuvant that recruits DCs to the vaccine site [19–21]. The MIP3α fused to gp100 then increases the efficiency of nascent vaccine protein uptake into infiltrating immature dendritic cells, resulting in enhanced downstream effector responses. This research provides further evidence for the utility of adding chemokine immunomodulators to vaccine constructs within any immunological context.

A primary strength of this DNA vaccine system is its modularity and ease of construction. This study shows that taking the gp100 antigen that induces a specific albeit not therapeutically relevant response on its own can become therapeutically relevant simply by fusing it to MIP3α. This observation raises the possibility that the response to more immunogenic antigens could be even further enhanced by the addition of MIP3α. A burgeoning new field in cancer vaccinology is the utilization of cancer-specific neoantigens as better vaccine targets that are not subject to T-cell central tolerance restrictions [3]. Our modular DNA vaccine could easily and rapidly be constructed to utilize neoantigens as they are discovered in real time. Testing the principle of this idea will be the subject of future studies, utilizing now delineated immunogenic neoantigens found in the B16F10 cell line [46]. In addition to neoantigens, future studies will also examine the efficacy of this vaccine system with other solid tumor models, in combination with current treatments such as immune checkpoint blockade, and in combination with novel immunomodulators.

## Conclusions

In conclusion, our data show that addition of MIP3α enhances the immunogenicity and efficacy of a therapeutic vaccine against the aggressive solid tumor, B16F10 mouse melanoma. The addition of MIP3α to therapeutic vaccines could present a useful strategy to enhance the responses of currently studied vaccines. Furthermore, the modularity of the plasmid provides a realistic platform for creating neoantigen vaccines in a clinically relevant time frame. These findings show that MIP3α can be a plug and play addition to the cancer immunologist’s vaccine toolbox that deserves further testing to determine the true potential of this novel design.

## Additional files

Additional file 1: Figure S1. Prophylactic vaccination protection confirmation. Mice were vaccinated three times over 2 week intervals with PBS or 50 μg MIP3α-gp100 by i.m. electroporation. Mice were challenged with a lethal dose of B16F10 (5 × 10^4^) 2 weeks after the third immunization. Tumor time course was tracked and analyzed by linear regression models. Tumor growth was found to be significantly reduced (*p* < 0.001), replicating prior published data. Data represent one experiment with 5–6 mice per group. (DOCX 97 kb)
Additional file 2: Figure S2. Vaccine peptide production in mammalian cell culture system. Different lanes represent different DNA preparations, with Mock being untransfected HEK-293 T cells. Weights in kDa of the ladder bands are noted. Full length construct is estimated to be 40 kDa, consistent with primary band below. (DOCX 169 kb)
Additional file 3: Dataset. Excel spreadsheet of dataset used in this publication. This file includes all of the data points shown in figures of this study. (XLSX 48 kb)

## Abbreviations

APC: Antigen presenting cell
B16F10: Mouse melanoma model
CSP: Circumsporozoite protein from malaria-causing parasite *Plasmodium falciparum*
DC: Dendritic cell
dMIP3α: Dysfunctional Macrophage Inflammatory Protein-3α. When utilized as a vaccine with gp100, it can be referred to as ‘antigen-only vaccine’
GP100: Glycoprotein 100, common melanoma differentiation antigen and vaccine target
MHC: Major histocompatibility complex
MIP3α: Macrophage Inflammatory Protein-3α
