# Supplementary material for: Fusion of the dendritic cell-targeting chemokine MIP3α to melanoma antigen Gp100 in a therapeutic DNA vaccine significantly enhances immunogenicity and survival in a mouse melanoma model
Source: J Immunother Cancer. 2016 Dec 20;4:96. doi: 10.1186/s40425-016-0189-y (PMC5168589; doi:10.1186/s40425-016-0189-y)
Supplement: Additional file 1: Figure S1. — Prophylactic vaccination protection confirmation. Mice were vaccinated three times over 2 week intervals with PBS or 50 μg MIP3α-gp100 by i.m. electroporation. Mice were challenged with a lethal dose of B16F10 (5 × 10^4^) 2 weeks after the third immunization. Tumor time course was tracked and analyzed by linear regression models. Tumor growth was found to be significantly reduced (p < 0.001), replicating prior published data. Data represent one experiment with 5–6 mice per group. (DOCX 97 kb) [file 40425_2016_189_MOESM1_ESM.docx]

Additional File 1

Prophylactic vaccination protection confirmation. Mice were vaccinated three times over two week intervals with PBS or 50μg MIP3α-gp100 by i.m. electroporation. Mice were challenged with a lethal dose of B16F10 (5x10^4^) two weeks after the third immunization. Tumor time course was tracked and analyzed by linear regression models. Tumor growth was found to be significantly reduced (p<0.001), replicating prior published data. Data represent one experiment with 5-6 mice per group.
