# Supplementary material for: Fusion of the dendritic cell-targeting chemokine MIP3α to melanoma antigen Gp100 in a therapeutic DNA vaccine significantly enhances immunogenicity and survival in a mouse melanoma model
Source: J Immunother Cancer. 2016 Dec 20;4:96. doi: 10.1186/s40425-016-0189-y (PMC5168589; doi:10.1186/s40425-016-0189-y)

Additional File 2:

Vaccine peptide production in mammalian cell culture system. Different lanes represent different DNA preparations, with Mock being untransfected HEK-293T cells. Weights in kDa of the ladder bands are noted. Full length construct is estimated to be 40 kDa, consistent with primary band below.


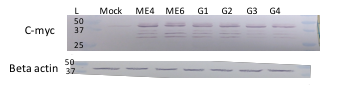

Supplement: Additional file 2: Figure S2. — Vaccine peptide production in mammalian cell culture system. Different lanes represent different DNA preparations, with Mock being untransfected HEK-293 T cells. Weights in kDa of the ladder bands are noted. Full length construct is estimated to be 40 kDa, consistent with primary band below. (DOCX 169 kb) [file 40425_2016_189_MOESM2_ESM.docx]
